# Estamos Chegando ao Fim do Caminho Evolutivo para Stents Metálicos Farmacológicos? Quais Stents de 4ª Geração Precisamos?

**DOI:** 10.36660/abc.20230302

**Published:** 2023-06-20

**Authors:** Manuel Sousa Almeida

**Affiliations:** 1 Centro de Intervenção Cardiovascular Estrutural Centro Hospitalar de Lisboa Ocidental Lisboa Portugal Centro de Intervenção Cardiovascular Estrutural – Centro Hospitalar de Lisboa Ocidental, Lisboa – Portugal; 2 NOVA Medical School Faculdade de Ciências Médicas Lisboa Portugal NOVA Medical School – Faculdade de Ciências Médicas, Lisboa – Portugal

**Keywords:** Stents/tendências, Anomalias dos Vasos Coronários, Intervenção Coronária Percutânea/tendências, Trombose Coronária, Angioplastia Transluminal Percutânea Coronária, Infarto do Miocárdio

Os stents são a pedra angular da PCI atual. Garantem segurança, resultado angiográfico agudo previsível e resultados clínicos aceitáveis a longo prazo.

Sua realização atual está, sem dúvida, ligada a um caminho evolutivo bem-sucedido impulsionado por contínuas necessidades médicas não atendidas enfrentadas por empresas de dispositivos ávidas por ganhar participação de mercado.

Desde o primeiro stent, objetivou-se o tratamento de salvamento de dissecções coronárias ameaçadoras,^1^ da trombose aguda de stent levantada como a complicação mais preocupante, posteriormente abordada com sucesso por aposição adequada da parede do stent^2^ e tratamento antiplaquetário duplo.^3^ Então, altas taxas de reestenose, particularmente em pessoas com diabetes, permaneceram como o maior obstáculo, resolvido em 2002 pelo advento dos stents farmacológicos^4^ acompanhados por trombose aguda tardia mais elevada,^5^ compensação devido a polímeros não biocompatíveis e cicatrização endotelial disfuncional.^6^ Inicialmente controlada com estratégias antiplaquetárias mais longas e agressivas^7^ e posteriormente resolvida com polímeros biocompatíveis mais recentes, os atuais stents de 3ª geração. No meio, novas ligas metálicas permitiram melhorias iterativas na espessura do suporte, flexibilidade, capacidade de entrega e conformabilidade do vaso, tornando o trabalho intervencionista muito mais fácil agora.^8^

Araujo et al.,^9^ em seu artigo intitulado: “ Avaliação no Mundo Real de um Stent Eluidor de Sirolimus de Hastes Ultrafinas em Pacientes com Infarto do Miocárdio com Supradesnivelamento do Segmento ST Submetidos à Intervenção Coronária Percutânea Primária (Registro INSTEMI)” analisou dados de um registro multicêntrico de STEMI do mundo real no sul do Brasil e comparou um novo stent ultrafino (75µm) de liga de CrCo com uma eluição de sirolimus de curto prazo (45 dias) de um polímero abluminal biocompatível degradável (9 meses) com uma mistura dos atuais stents workhorses padrão.

Para esta comparação, eles selecionaram de uma população maior de pacientes com STEMI tratados com PPCI em dois centros terciários, dois grupos pareados por propensão, cada um com 353 pacientes, tratados com stent Inspiron™ (709 stents) e um grupo controle, uma mistura de DES , quanto ao fármaco (sirolimus em 22% e não sirolimus em 78%), distribuição do polímero biodegradável (abluminal em apenas 2%) e durabilidade (durável em 78%) e espessura do suporte (≤75µm em 20%), em um total de 716 stents de stents atuais de 3ª geração. Embora tal heterogeneidade, para este estudo, não compromete os resultados e conclusões. Como afirmado, os autores tiveram como objetivo comparar um DES mais recente com os DES workhouse atuais em um padrão de atendimento do mundo real.

Além das apontadas pelos autores, teve algumas limitações como a duração do 2DPT, não mencionada em nenhum lugar, por protocolo a critério do operador. Diferenças entre os grupos poderiam comprometer os resultados em um acompanhamento tão longo. Apesar dos grupos de propensão combinada, o comprimento do stent foi significativamente maior no Inspiron™ (10 mm mais longo em média), juntamente com outras pequenas diferenças também consideradas.

No entanto, e para este estudo, eles chegaram a conclusões válidas: até 17 meses de seguimento, em pacientes tratados por PPCI, o uso do stent Inspiron™ parece ser tão seguro e eficaz quanto outros stents de terceira geração padrão de tratamento atuais.

Um copo meio cheio, meio vazio. O Inspiron™ é tão bom quanto os stents atuais, mas não é melhor! Tenho dúvidas se comparar um número maior de pacientes resolverá a questão, já que os resultados clínicos se tornaram tão semelhantes e tão baixos, questionando se é possível ir além.

Outras questões, como capacidade de entrega e acesso aprimorado ao ramo lateral, também são valiosas para os cardiologistas intervencionistas. Um stent não colocado corretamente ou que impeça o acesso a um ramo lateral pode comprometer todos os procedimentos. Tais “detalhes” não são comumente captados na maioria dos estudos. Além da intenção de tratar todos os participantes, ensaios randomizados, registros e pequenos estudos não são desenvolvidos e projetados para abordar adequadamente esses “detalhes”. Ele vem com a experiência diária individual.

Um stent ultrafino com menos metal, uma geometria adequada e um bom compromisso entre força radial, flexibilidade, conformabilidade e capacidade de entrega pode se tornar uma quarta geração. No entanto, suponha que almejamos melhorias futuras significativas. Nesse caso, precisamos estender para outras plataformas de stent, o que garante segurança com menos 2DAPT e entregabilidade, desaparecendo quando o andaime não é mais necessário, restaurando a normalidade das paredes dos vasos e prevenindo a neoaterosclerose local. Por que não?
