# Supplementary material for: Estamos Chegando ao Fim do Caminho Evolutivo para Stents Metálicos Farmacológicos? Quais Stents de 4ª Geração Precisamos?
Source: Arq Bras Cardiol. 2023 Jun 20;120(6):e20230302. [Article in Portuguese] doi: 10.36660/abc.20230302 (PMC10344366; doi:10.36660/abc.20230302)
Supplement: Translation

# Translation: Are We Reaching the End of the Evolutionary Road to Metallic Drug-Eluting Stents? Which 4^th^ Generation Stents Do We Need?

**Authors:** Manuel Sousa Almeida

**Affiliations:** 1 Portugal Centro de Intervenção Cardiovascular Estrutural – Centro Hospitalar de Lisboa Ocidental, Lisboa – Portugal; 2 Portugal NOVA Medical School – Faculdade de Ciências Médicas, Lisboa – Portugal

**Keywords:** Stents/trends, Coronary Vessels Anomalies, Percutaneous Coronary Intervention/trends, Coronary Thrombosis, Angioplasty,Transluminal Percutaneous Coronary, Myocardial Infarction

Stents are the cornerstone of current PCI. It guarantees safety, a predictable acute angiographic upshot, and long-term acceptable clinical outcomes.

Its current accomplishment is, undoubtedly, linked to a successful evolutionary road driven by continuous unmet medical needs endured by device companies eager to gain market shares.

From the first stent, aimed to bailout treatment of threatful coronary dissections,^1^ acute stent thrombosis upraised as the most concerned complication, later successfully addressed by proper stent wall apposition^2^ and double antiplatelet treatment.^3^ Then, high rates of restenosis, particularly in diabetics, remained the biggest restraint, who was solved in 2002 by the advent of drug-eluting stents^4^ accompanied by higher late acute stent thrombosis,^5^ a trade-off due to non-biocompatible polymers and dysfunctional endothelial healing.^6^ Initially, controlled with longer and more aggressive antiplatelet strategies^7^, was later resolved with newer biocompatible polymers, the current 3rd generation stents. In between, newer metallic alloys allowed iterative improvements in strut thickness, flexibility, deliverability, and vessel conformability, making interventionist work much easier now.^8^

Araujo et al.,^9^ in their paper entitled: “Real-World Assessment of an Ultrathin Strut, Sirolimus-Eluting Stent in Patients with ST-Elevation Myocardial Infarction Submitted to Primary Percutaneous Coronary Intervention (INSTEMI Registry)”, analyzed data from a real-world STEMI multicenter registry in southern Brazil, and compared a new ultrathin (75µm) CrCo alloy stent with a short term sirolimus elution (45 days) from a biocompatible degradable (9 months) abluminal polymer with a mix of current standard workhorses stents.

For this comparison, they select from a larger population of STEMI patients treated with PPCI in two tertiary centers, two propensity-matched groups, each with 353 patients, treated with Inspiron™ stent (709 stents) and a control group, a mix of DES, regarding the drug (sirolimus in 22% and non-sirolimus in 78%), the biodegradable polymer distribution (abluminal in only 2%) and durability (durable in 78%), and the strut thickness (≤75µm in 20%), on a total of 716 stents of current 3^rd^ generation stents. Although such heterogeneity, for this study, it does not compromise the results and conclusions. As stated, the authors aimed to compare a newer DES with the current workhorses DES in a real-world standard of care.

In addition to those pointed out by the authors, it had some limitations, such as the duration of the 2DPT, not mentioned anywhere, per protocol at the operator’s discretion. Differences between groups could jeopardize the results in such a long follow-up. Albeit the propensity-matched groups, stent length was significantly and meaningful higher in Inspiron™ (10mm longer on average), along with other minor differences also accounted for.

Nevertheless, and for this study, they reached valid conclusions: until 17 months follow-up, in patients treated by PPCI, the use of Inspiron™ stent seems to be as safe and effective as other current standard-of-care third-generation stents.

A glass half full, half empty. The Inspiron™ is as good as current stents, but not better! I am doubtful if comparing a larger number of patients will resolve the question, as clinical outcomes became so similar and so low, questioning if it is possible to go further.

Other issues like deliverability and enhanced side branch access are also valuable to interventional cardiologists. A stent not properly delivered or impeding a side branch access may jeopardize all procedures. Such “details” are not commonly grabbed in most studies. Besides the intention to treat all comers, randomized trials, registries, and small studies are not powered and designed to properly tackle such “details.” It comes with individual daily experience.

An ultrathin stent with less metal, a proper geometry, and a good compromise between radial force, flexibility, conformability, and deliverability may become a fourth generation. However, suppose we aim for meaningful future improvements. In that case, we need to extend up for other stent platforms, which guarantees safety with less 2DAPT and deliverability, vanishing a way when scaffolding is no longer needed, restoring vessel walls to normality, and preventing local neo-atherosclerosis. Why not?
